# Selecting Training-Load Measures to Explain Variability in Football Training Games

**DOI:** 10.3389/fpsyg.2019.02897

**Published:** 2020-01-24

**Authors:** Unai Zurutuza, Julen Castellano, Ibon Echeazarra, Ibai Guridi, David Casamichana

**Affiliations:** ^1^Physical Education and Sport Department, Faculty of Education and Sport, University of the Basque Country – UPV/EHU, Vitoria-Gasteiz, Spain; ^2^Physical Performance Department, SD Beasain, Beasain, Spain; ^3^Faculty of Physiotherapy and Speech Therapy Gimbernat-Cantabria University School Associated with the University of Cantabria, Torrelavega, Spain

**Keywords:** team sport, time motion, heart rate, small-sided game, principal component analysis

## Abstract

The purpose of this study was to investigate the structure of interrelationships among external (eT) and internal (iT) training intensity metrics and how these vary depending on game format in soccer. The variables were collected from 16 semi-professional players in seven types of small, medium, large-sided, and simulated games (SG). The eT variables were (per min): peak velocity (*V*max), total distance (DTmin), distance covered at velocities less than 60% (*D* < 60%min), between 60 and 80% (*D* > 60%min), and more than 80% (*D* > 80%min) of the maximal velocity, player load (PLmin), and distance covered accelerating at more than 2 m⋅s-2 (Daccmin) and decelerating at less than −2 m⋅s-2 (Ddecmin). The iT variables were: Edwards arbitrary units (EDWmin) and time spent at more than 80% of the maximal heart rate (*T* > 80% HRmin). All game formats were represented by three principal components (PC), explaining from 66.9 to 76.0% of the variance. The structure of the interrelationships among variables involved similar distributions in the PCs that are related to energetic production systems, such as the strength/neuromuscular dimension (PLmin and/or Daccmin and Ddecmin, complemented by DTmin and *D* < 60%min), the endurance/cardiovascular dimension (EDWmin), and the velocity/locomotion dimension (*V*max, *D* > 60%min, or *D* > 80%min). A particular combination of external and internal intensity measures is required to describe the training load of game formats.

## Introduction

Research using a mixed-methods approach presents us with the challenge of combining and integrating quantitative and qualitative data in the same study (Anguera et al., 2018). Although this approach is not new, it continues to attract increasing attention. Recently, it has been applied in sports contexts in order to explain the behavior of football players (Maneiro and Amatria, 2018; Maneiro et al., 2019) or teams (Diana et al., 2017).

Football players are subject to different types and amounts of load during training sessions, with the aim of optimizing their performance (Graham et al., 2018) in competition and reducing, as far as possible, the risk of injury (Gabbett, 2016). For this reason, monitoring the training load in a systematic way is a key aspect in being able to plan and intervene on the quantity, quality, and appropriate order of the training process, with the aim of maximizing its efficiency (Impellizzeri et al., 2005). The evaluation of the training load in general, and specifically that of the underlying training tasks, is indispensable both in terms of optimizing the players’conditional performances (avoiding under- or over-training through conditions that are very different from those of matches) and in preventing overtraining and injuries (Sangnier et al., 2018).

Reduced games are sport motor situations (Parlebas, 2001) that include most of the factors that arise in the “real” game in an adaptable way (Renshaw et al., 2009). An important part of the content of football training is related to the tasks performed, e.g., small- (SSG), medium- (MSG), and large-sided games (LSG) (Little, 2009). The pitch dimensions of interaction in the task proposals affect the quality and quantity of the driving behavior of players, in which significant differences have been detected in the game. The individual space of interaction (ISI) is an important variable to consider in the design of tasks for training in soccer. There is extensive literature supporting the hypothesis that different game formats demand particular patterns of movement, provoking a specific response in players and having acute and chronic effects on physical condition (Hill-Haas et al., 2011; Aguiar et al., 2013; Sgrò et al., 2018). Nevertheless, due to the multiple conditional demands of SSGs, there is no consensus as to what variables better represent those demands.

Recent research studies (Weaving et al., 2017a, b, 2018) support the idea that a single training load variable is not sufficient to capture a significant proportion of the variety provided by multiple load variables. For this reason, it is usually decided to use a multitude of variables, e.g., global load indicators or intensity variables, to describe the demands and the response provoked in the players during training and/or competition (Akenhead and Nassis, 2016). However, managing a multitude of variables is not very efficient (provoking, in some cases, redundancy in the information reported), making it difficult for the physical practitioner to carry out a thorough follow-up and control of the stimulus provoked to the players, which makes it necessary to use strategies that allow the management of the minimum amount of variables necessary to have the essential information (Williams et al., 2016).

With the aim of reducing redundancy (Casamichana et al., 2019), one of the strategies recently proposed in rugby (Weaving et al., 2017a) for workload monitoring of different training modes, or in basketball (Casamichana and Castellano, 2015) for comparing the differences among players’ positions, is related to with the implementation of principal component analysis (PCA). The use of this analytical technique allows it to be determined whether we are using redundant information, e.g., variables that provide the same information about the load or the intensity implied by the practice of the tasks performed (Casamichana and Castellano, 2015).

Principal component analysis is based on a systematic process that allows a reduction in the number of variables to attend to, minimizing the loss of information associated with that process. Currently, more information is required in order to determine how physical and physiological variables are related in different game formats and in simulated games (SG) so as to allow a fine-tuning of the selection of variables necessary to provide all the information needed by preparers to design and control the stimuli demanded from the players.

In this way, the purpose of the current study was to investigate the structure of interrelationships between the external and internal training intensity variables and determine how they vary between different types of SSG-, MSG-, and LSG (e.g., from 3 vs. 3 to 10 vs. 10). Considering hypothetical results, if different training tasks involve no similar physical demands, it could help coaches to design tasks where players can replicate the demands that will probably be required during games.

## Materials and Methods

### Participants

A total of 23 semi-professional male football players from group IV of the third division in the Spanish League took part in the study. Due to the number of devices available, information related to 16 of them was used (age = 25.1 ± 3.7 years; height = 178.3 ± 5.0 cm; weight = 74.6 ± 7.9 kg; percentage of body fat obtained with the Möhr formula = 10.8 ± 2.2%). The players completed, on average, 3–4 weekly training sessions and played one official match every weekend. Before taking part in the study, all the players involved signed an informed consent form. Participants and the team’s technical staff were informed about the procedure and possible risks and benefits of the study. Furthermore, the procedures used in this project were in accordance with the Declaration of Helsinki and the Ethics Committee of the University of the Basque Country (UPV/EHU), which also gave its institutional approval of the study.

### External Intensity Variables

The following external variables were studied: peak velocity (*V*max), total distance (DTmin), distance covered at less than 60% (*D* < 60%min), between 60 and 80% (*D* > 60%min) and more than 80% (*D* > 80%min) of the maximal velocity of each player, player load (PLmin), and distance covered accelerating at more than 2 m⋅s-2 (*D*acc > 2min) and decelerating at less than −2 m⋅s-2 (*D*dec < −2min). Except for the variable *V*max, the rest of the measures were relativized and expressed in minutes of practice. All of these external variables are related to the locomotor (distance and velocity) and neuromuscular (acceleration/deceleration) dimensions.

### Internal Intensity Variables

In all training sessions, HR was recorded with a short-range telemetry system (Polar Team2 Pro System, Polar Electro Oy, Kempele, Finland). The Edwards method was used (EDWmin) to quantify the internal load from the HR (Edwards, 1993). This method distributes the magnitude of the HR in five different zones. Each zone has a value associated with it (50–60% HRmax = 1, 60–70% HRmax = 2, 70–80% HRmax = 3, 80–90% HRmax = 4, and 90–100% HRmax = 5), and these are later added together. The second variable was time spent at more than 80% of the maximal HR (*T* > 80%HRmin), similar to that proposed by Henderson et al. (2015).

To calculate the maximum HR for each player, a maximal progressive test was carried out on a treadmill with a HR monitor, beginning at a velocity of 8 km⋅h^–1^ and increased at a rate of 1 km⋅h^–1^ every minute until the point of physical exhaustion was reached (Graff, 2002). All HR-based measures are related to the endurance dimension.

### Assessment of Small-Sided, Medium-Sided, Large-Sided, and Simulated Games

Eight game formats were used in this study, involving different numbers of players per team, always with a goalkeeper and official football eleven-a-side goals. The players did not have any technical or tactical limitations during the performance of the SSG. Considering the duration of the tasks, number of bouts, and dimensions of the field, the eight game formats were grouped into four types of training tasks: 3 vs. 3 as SSG, 4 vs. 4, 5 vs. 5, and 6 vs. 6 as MSG, 7 vs. 7, 8 vs. 8, and 9 vs. 9 as LSG, and 10 vs. 10 as SG, with the constraints that appear in Table 1.

**TABLE 1:** Description of the features of the four groups of training tasks: small-sided games (SSG), medium-sided games (MSG), large-sided games (LSG), and simulated games (SG).

Format	Players per team (*n*)	Records (*n*)	Pitch size (m^2^ per player)	Number of bouts (*n*)	Duration of bouts (min:sec)
SSG	3	25	≈ 84	4	≈ 3:30
MSG	4	216	≈ 132	3	≈ 3:00
	5	238	≈ 105	4	≈ 5:00
	6	28	≈ 130	4	≈ 6:30
LSG	7	26	≈ 247	2	≈ 17:00
	8	60	≈ 272	3	≈ 13:00
	9	44	≈ 235	2	≈ 15:00
SG	10	61	≈ 300	2	≈ 19:00

### Procedures

This observational study was carried out during seven consecutive microcycles (from 30 to 37) of a competitive period (from February to April) of the 2016–2017 season. The specific observational design (Anguera et al., 2011) employed was: point (without intersessional follow-up), multidimensional (analysis of internal and external load), and nomothetic (focus on several players).

Heart rate sensors and GPS devices monitored all training sessions. Before beginning the study, the players underwent a maximal progressive resistance test on a treadmill (in a laboratory) to calculate the maximum heart rate (HR) of each player and a 40-meter velocity test on the training ground provided whilst wearing the GPS devices to measure the individual *V*max (Roe et al., 2017). In total, 698 recordings were collected in 16 training sessions (43.6 ± 12.1 per player). The quality of the signal of the GPS devices was assessed: the mean (± sd) number of satellites during data collection was 12.5 (± 0.6) (Castellano et al., 2011).

Physical demands were measured using a portable GPS device operating at a sampling frequency of 10 Hz, which contains a 100 Hz triaxial accelerometer (Minimax v.4.0, Catapult Innovations Victoria, Australia). The device was attached to the upper back of each player using a special harness. The GPS devices were activated 15 min before the start of each session or match, in accordance with the manufacturer’s instructions. Collected data from the Minimax S4 and PolarTeam2 devices were downloaded to a PC to be analyzed using the Sprint v5.1.4 software package (Catapult Innovations, Victoria, Australia, 2010).

The validity and reliability of this technology have been previously demonstrated, indicating that it is a valid way of monitoring different speed ranges (Johnston et al., 2014). The internal response was assessed based on HR (Alexandre et al., 2012), which was recorded every 5 s using a telemetric device (Polar Team Sport System, Polar Electro Oy, Finland). All the players were familiarized with the use of both GPS and HR monitors before starting the study.

### Statistical Analysis

Descriptive statistics data from the training games are reported as the mean and standard deviation (± sd). Additionally, magnitude-based inferences were used to analyze the data based on the recommendations of Batterham and Hopkins (Batterham and Hopkins, 2006). Differences between SSG, MSG, LSG, and SG were assessed via standardized mean differences (Cohen’s d with a 90% confidence limits). The interpretation thresholds for standardized effect size (ES) were as follows (Batterham and Hopkins, 2006): <0.2 (trivial), 0.2–0.6 (small), 0.6–1.2 (moderate), 1.2–2.0 (large), and >2.0 (very large).

Before carrying out PCA, the Pearson correlation matrix with ten external and internal training intensity variables was constructed in order to perform a visual inspection of data factorability (Tabachnick and Fidell, 2007). This method aims to extract the most important components and/or variables from data without reducing the volume of information. The Kaiser-Meyer-Olkin (KMO) method was used to verify whether the 10 external load variables were suitable for PCA, i.e., KMO > 0.5 (Kaiser, 1960). The KMO values for the four game formats were 0.54, 0.516, 0.514, and 0.522 for SSG, MSG, LSG, and SG, respectively, showing that the dataset is suitable for PCA. Bartlett’s sphericity test was significant for each training mode (*p* < 0.001). The principal axis method was used to extract the components. Components with eigenvalues of less than 1 were not retained for extraction (Kaiser, 1960).

The PCA was applied with a VariMax rotation to identify components that are not highly correlated. Subsequently, the rotation was performed with the goal of making the 9 × 1 component loadings more easily interpretable. The stages involved in the calculation for PCA were the same as those used previously (Weaving et al., 2014). Following the methods of Weaving and colleagues (Weaving et al., 2014) for each extracted PC, only the original variables that possessed a PC loading greater than 0.7 were retained for interpretation.

Finally, the correlation between external and internal load variables was measured for each game format. Following Hopkins, the following qualitative correlation descriptors were used: trivial (0–0.09), small (0.1–0.29), moderate (0.3–0.49), large (0.5–0.69), very large (0.7–0.89), nearly perfect (0.9–0.99), and perfect (1) (Hopkins, 2000). The Statistical Package for the Social Sciences (SPSS, Version 24.0 for Windows; SPSS Inc., Chicago, IL, United States) and JASP version 0.7.5 (Love et al., 2015) were used to conduct the analysis.

## Results

The means and standard deviations of each physical measure and HR derived variable recorded in the eight types of format games are shown in Table 2. There were significant differences among game formats, specifically: larger game formats had higher velocity demands (maximal and average), while smaller formats demanded more acceleration and deceleration. The demands derived from HR were higher in the SSG and MSG compared to the LSG and SG.

**TABLE 2:** Means and standard deviations (± sd) of internal and external training intensity measures according to the groups of game formats.

Load measures	Variables (units)	SSG	MSG	LSG	SG
External (eTL)	*V*_max_ (km⋅h^–1^)	17.9 ± 2.8	18.9 ± 2.7	24.0 ± 2.7	25.1 ± 2.2
	*D* < 60%min (m⋅min^–1^)	94.2 ± 16.9	96.6 ± 14.4	99.0 ± 14.8	100.3 ± 13.8
	*D* > 60%min (m⋅min^–1^)	1.6 ± 2.5	3.2 ± 3.7	5.0 ± 2.8	6.2 ± 2.5
	*D* > 80%min (m⋅min^–1^)	0.0 ± 0.0	0.1 ± 1.0	0.6 ± 1.0	1.2 ± 1.3
	DTmin (m⋅min^–1^)	95.8 ± 18.0	100.1 ± 15.5	104.7 ± 16.0	107.6 ± 14.4
	PLmin (AU⋅min^–1^)	11.9 ± 2.9	11.2 ± 2.4	9.8 ± 2.0	10.0 ± 1.9
	*D*acc > 2min (m⋅min^–1^)	4.6 ± 1.3	4.4 ± 1.6	3.6 ± 1.0	3.4 ± 0.9
	*D*dec < −2min (m⋅min^–1^)	3.1 ± 1.3	3.1 ± 1.3	2.8 ± 0.9	2.6 ± 0.8
Internal (iTL)	EDWmin (AU⋅min^–1^)	3.3 ± 1.5	3.6 ± 1.0	3.1 ± 1.0	3.2 ± 0.7
	*T* > 80%HRmin (min⋅min^–1^)	0.3 ± 0.4	0.7 ± 1.2	0.3 ± 0.5	0.2 ± 0.4

*SSG is 3 vs. 3 (three players per team), MSG involves 4 vs. 4, 5 vs. 5, and 6 vs. 6 game formats, LSG includes 7 vs. 7, 8 vs. 8, and 9 vs. 9 game formats, and SG is a simulated game (10 vs. 10). Vmax is peak velocity, DTmin is total distance covered, *D* < 60%min is distance covered at less than 60% of the maximal velocity of each player, *D* > 60%min is distance covered at between 60 and 80% of the maximal velocity of each player, *D* > 80%min is distance covered at more than 80% of the maximal velocity of each player, Mmin is distance covered per minute, EDWmin is Edwards arbitrary units per min, *T* > 80%HRmin is time spent at more than 80% of the maximal heart rate, PLmin is player load per minute, *D*acc > 2min is distance covered accelerating at more than 2 m⋅s^–2^, and *D*dec < −2min is distance covered decelerating at less than −2 m⋅s^–2^.*

**FIGURE 1:**
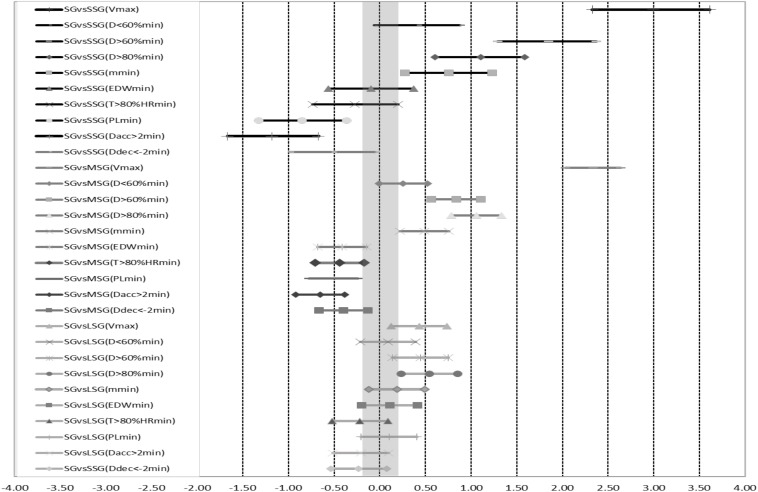
Effect sizes for the simulated games (SG) format compared with the other three, small-sided (SSG), medium-sided (MSG), and large-sided (LSG) games. *V*_*max*_ is peak velocity, DTmin is total distance covered, *D* < 60%min is distance covered at less than 60% of the maximal velocity of each player, *D* > 60%min is distance covered at between 60 and 80% of the maximal velocity of each player, *D* > 80%min is distance covered at more than 80% of the maximal velocity of each player, EDWmin is Edwards arbitrary units per min, *T* > 80%HRmin is time spent at more than 80% of the maximal heart rate, PLmin is player load per minute, *D*acc > 2min is distance covered accelerating at more than 2 m⋅s^–2^, and *D*dec < –2min is distance covered decelerating at less than –2 m⋅s^–2^.

Figure 1 represents ES for the SG format compared with the other three game formats. At the bottom of the figure, it can be observed that SG and LSG do not differ substantially in any of the compared variables. All of the variables analyzed showed small magnitude differences between SG and LSG, but the differences become higher and lower (depending on the assessed variable) when compared to SSG or MSG. Variables involving a velocity dimension (e.g., *V*max, *D* > 60%min, *D* > 80%min and DTmin) were higher in SG with a moderate to very large effect, while variables regarding strength (e.g., PLmin, *D*acc > 2min and *D*dec < −2min) were higher in SSG and MSG with a small to moderate effect.

**FIGURE 2:**
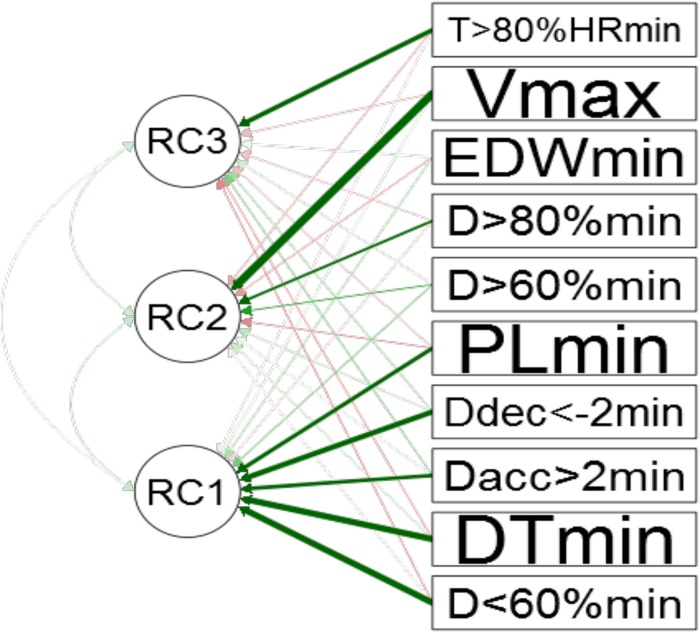
Results of the PCA, showing the rotated training load component loadings for each PC extracted (values above 0.7 are highlighted: in PC1, *D* < 60%min = 0.83, PLmin = 0.74, DTmin = 0.86, *D*acc > 2min = 0.79, and *D*dec < −2min = 0.84, in PC2, *V*max = 0.82 and *D* > 80%min = 0.72, and in PC3, *T >* 80%HRmin = 0.79).

**FIGURE 3:**
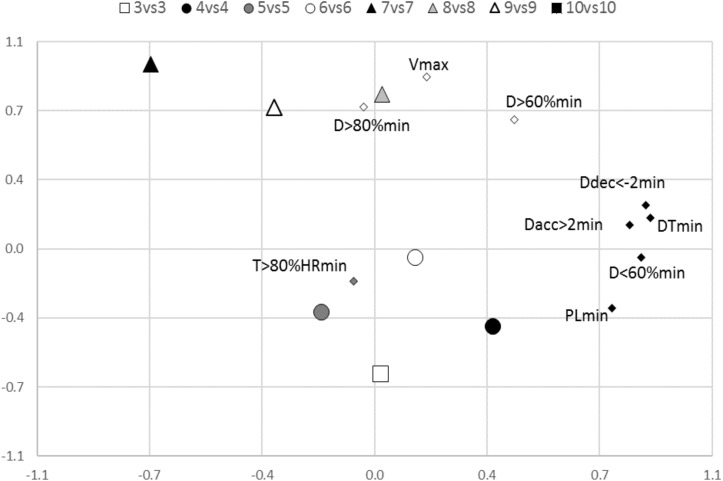
Game format distribution as the average position of each training task through the two principal components (first and second). The game formats are from 3 vs. 3, the small-sided game with three players per team, to 10 vs. 10 or simulated games.

Regarding PCA (Figure 2), the eigenvalues for each principal component were 3.79, 1.82, and 1.05 for the first (PC1), second (PC2), and third (PC3) principal components, respectively. The total explained variances by each principal component were: 37.90, 18.24, and 10.53 for PC1, PC2, and PC3, respectively. Figure 3 shows the representativeness of the 10 iTL and eTL intensity variables (rotated component).

Finally, Figure 3 shows the position of each game format in the rotated component plot. Only two main factors were plotted so as to visually represent the differences between game formats.

## Discussion

The aim of this research was to study the identification of a structure with three principal components summarizing eight external and two internal intensity variables for all of the types of game formats studied. This is the first piece of work that focuses on assessing the demands and responses of the same team in eight different game tasks grouped into four types of game format (SSG, MSG, LSG, and SG). The main value of this study is the opportunity to compare players of the same team in different game formats that are usually practiced in football training sessions, understanding the characteristics associated with each of the formats. The main results of the study can be summarized as follows: (1) through PCA analysis, determine the minimum amount of variables necessary to obtain the essential information and (2) thus obviate redundant information in workload analysis and help to save effort on the part of physical trainers and increase the quality of their analyses.

The application of this procedure to determine the minimum amount of variables can be applied to load adjustment for each of the variables. This method (PCA) aims to extract the most important components and/or variables from data without reducing the information. Although the initial number of factors was the same as the number of variables used in the factor analysis, only the first three (1, 2, and 3) PC were retained in the present study. The total percentage of variance explained by the sum of the three rows (factors) used was 66.7%. C1 involved five eTLs, namely *D* < 60%, PL, DT, *D*acc > 2, and *D*dec < −2, C2 was represented by three variables, namely *V*max, *D* > 60%, and *D* > 80%, and, finally, only one iTL had a score above 0.70 (all values were relative to minutes of practice). Considering this, we can conclude that these factors (depending on game format) adequately represent the demands and responses in the original data.

Firstly, from a comparative point of view, the demands and responses associated with the different groups of game formats when compared to simulated games (SG) follow the same profile found by previous studies. Larger field dimensions and a higher number of players per team translates into higher demands in terms of *V*max and *D* > 80%min (Casamichana and Castellano, 2010; Hodgson et al., 2014). Conversely, when both variables (dimensions and players) are lower, more demand is placed on acceleration and deceleration variables (Castellano and Casamichana, 2013; Castellano et al., 2015).

In almost all of the variables (except for *D* < 60%min, *T* > 80%min, and EDWmin), the differences between the extreme formats, e.g., SSG versus SG, are from moderate to large. However, between more similar training game formats, e.g., MSG versus SSG, these differences become small or trivial. As has been suggested previously (Casamichana et al., 2019), the lack of similarity between the demands of the four groups of training formats could suggest the need to use the whole range of training game formats (e.g., from 1 vs. 1 to 10 vs. 10) when coaches want to overstimulate or replicate the demands of competition (SG in the current study), having as a reference the particular needs of each playing position (Delaney et al., 2018; Lacome et al., 2018).

The first principal component explained the greatest proportion of variance (38%), involving five of the ten variables studied. Three out of the five external training load variables involved in this component (PL, ACC, and DEC) have a close relation to the neuromuscular or strength dimension. Furthermore, DTmini and distance covered above 60% of the individual velocity are also representative of this component. Previously, it has been shown that there is a high correlation between PL and DT in both training sessions (Casamichana et al., 2013) and training tasks (Casamichana and Castellano, 2015). According to the academic literature (Castellano and Casamichana, 2013; Hodgson et al., 2014), SSG and MSG request more intermittent activity in players, with less time in recovery periods (*D* < 60%min) and more PLmin.

In relation to the second component, *V*max and DT > 80%min had the most representativeness. These two variables are related to the locomotor or velocity dimension. As can be seen in Figure 3, game formats with higher dimensions, numbers of players per team, and durations of the activity are the ones that plot closer to this component. Once again, this is in line with the previous proposals (Casamichana et al., 2019) in relation to the type of training formats that replicate football-eleven velocity demands. The higher the dimensions of the field, the greater the demands related to high running velocity (e.g., peak velocity and/or distance accumulated running at high speed) (Casamichana and Castellano, 2010; Casamichana et al., 2019).

Finally, the third component was represented by the variables related to HR measures, which involved an endurance dimension. Even if EDWmin (as the global internal indicator) did not have any weight in this dimension, *T* > 80%HRmin was the iTL variable that best represented it. The training formats closer to this variable were SSG and MSG, which means that with a reduced number of players per team, they become more directly involved in the game. These results are consistent with those reported by other studies of SSGs in soccer (Brandes et al., 2011), being an effective means of improving endurance in soccer players (Dellal et al., 2008).

With respect to the identification of a structure, we conclude that all game formats could be represented by three dimensions (e.g., cardiovascular, locomotor, and neuromuscular), all of which are necessary to categorize the spectrum of demands on and responses of players in the range of side games in football. Analyzing the three dimensions and determining the variables needed for one correct and high-quality analysis of the workload would be sufficient.

A lack of inclusion of additional variables of the game formats studied in the analysis (e.g., number of bouts, duration and type of rest periods, etc.) is one of the limitations of the present study. It is possible that these variables could affect the results obtained. Different distributions of the activity durations and recovery periods of the game formats could have made specific demands. The second limitation involves the differentiation between playing positions (Casamichana et al., 2019) or even between players (Weaving et al., 2017a). In those cases, other factors and correlations between variables could emerge. Consequently, further research is required to establish the demands and responses associated with different game formats in relation to specific playing positions and/or individual players.

The results obtained in the present study provide very interesting findings. Firstly, they show that a combination of external and internal intensity variables explains a high proportion of the variance observed in the training game formats performed by a semi-professional football team (e.g., from 3 vs. 3 to 10 vs. 10 plus goalkeeper). Secondly, they indicate that when the same players participate in different game formats, the demands of the training tasks are not equal. For this reason, it could be interesting to consider different types of game format depending on the conditional objective of the session in order to replicate, overload, or underload the game demands (Casamichana et al., 2019). In any case, it seems interesting to include variables from different dimensions in the load management process, with the objective of assessing with accuracy the demands and responses invoked by the training formats used. As presented throughout the paper, each game format represents/involves specific demands and responses but with a similar structure of dimension demanded (e.g., the same dimension but with a different weight for each variable).

### Practical Applications

The findings of this study focus on the demands of different training game formats and how a reduced number of variables can be selected while keeping the maximum amount of information, providing coaches with information with which to enhance the effectiveness of the design and assessment of training sessions and weekly periodization. A combination of internal and external intensity variables allows a deep description of the current demands of and responses to game formats that are usually applied by coaches in daily training sessions. Using all of those game formats integrates the majority of requirements that are placed on players when competing. Once coaches consider the different demands of and responses to all the variety of game formats in football (e.g., from 1 vs. 1 to 10 vs. 10), optimal training loads can be proposed, overloading or under-loading depending on the necessity of the moment in the session, week, or in a larger periodization.

## Conclusion

The conclusion of the study was that a combination of different game formats explained all the variables that have been analyzed in the present study. The authors agree with the suggestion of previous research studies (Casamichana et al., 2019) that confirm the idea that different types of stimulus are necessary to optimize the conditional demands on players. The different training game formats used showed that the acceleration and deceleration component was the most stimulated in SSG, the cardiovascular demands were highest in MSG, and peak and average velocity were most demanded in LSG and SG. Future research should focus on the study of this type of different game format analysis with regards to player positions and/or individual profiles.

## Data Availability Statement

The raw data supporting the conclusions of this article will be made available by the authors, without undue reservation, to any qualified researcher.

## Ethics Statement

The studies involving human participants were reviewed and approved by M10/2015/303/CASTELLANO PAULIS. The patients/participants provided their written informed consent to participate in this study.

## Author Contributions

UZ and JC: conceptualization, investigation, and resources. UZ, IE, IG, and JC: methodology and writing – original draft preparation. UZ: data extraction. JC: analysis and supervision. UZ, JC, and DC: data management. IE, IG, and DC: writing – review and editing.

## Conflict of Interest

The authors declare that the research was conducted in the absence of any commercial or financial relationships that could be construed as a potential conflict of interest. The handling Editor declared a past co-authorship with one of the authors JC.
